# Skin Metastasis Occurring 30 Years After Thyroidectomy for Papillary Thyroid Carcinoma

**DOI:** 10.7759/cureus.22180

**Published:** 2022-02-13

**Authors:** Mohammed S Alwhaid, Olaa Mhish, Mutahir A Tunio, Salman AlMalki, Mushabbab Al Asiri, Khalid Al-Qahtani

**Affiliations:** 1 Radation Oncology, King Faisal Specialist Hospital & Research Centre, Riyadh, SAU; 2 Faculty of Medicine, Alfaisal University College of Medicine, Riyadh, SAU; 3 Radiation Oncology, King Fahad Medical City, Riyadh, SAU; 4 Histopathology, King Fahad Medical City, Riyadh, SAU; 5 Otolaryngology, King Saud University, Riyadh, SAU

**Keywords:** treatment, diagnosis, papillary thyroid carcinoma, metastasis, skin

## Abstract

The skin is an extremely rare site of metastasis from papillary thyroid carcinoma (PTC) and is linked to underlying disseminated malignancy, which reflects a dismal prognosis. We present the case of a 70-years-old Saudi female who presented at our clinic with an eight-month history of two painful and itchy skin nodules over the scalp and the medial aspect of the right arm. She had a history of total thyroidectomy for PTC 30 years prior. Computed tomography-positron emission tomography showed multiple fluorodeoxyglucose avid lung and skeletal metastases. This case highlights the fact that skin nodules in a patient with a history of PTC should be assessed carefully with a high suspicion of skin metastasis to avoid any delay in treatment.

## Introduction

Thyroid carcinoma is the second most common malignancy following breast cancer among females in Saudi Arabia [1]. Despite the excellent prognosis of the papillary thyroid carcinoma (PTC) type, approximately 4-23% of patients with PTC eventually progress to metastatic disease, predominantly to the lungs, bone, and central nervous system [2,3]. Skin as a site of distant metastasis secondary to PTC is an extremely rare entity (<1%) [4]; however, the scalp and upper torso are the most common locations [5]. In our previous study of 370 Saudi patients with PTC treated at our hospital, distant metastases manifested only in 10.4% of the cases, with no single case with skin as a site of distant spread [6]. Furthermore, cutaneous metastases are known to be associated with poor prognosis secondary to a huge underlying disease burden, with an average life span of eight to 19 months [7]. Here, we present a rare case of skin metastasis to the scalp and right medial arm that occurred 30 years after the primary treatment for PTC.

## Case presentation

A 70-years-old non-smoking Saudi female without a family history of thyroid cancer presented to our clinic with complaints of mildly painful skin nodules over the scalp and an itchy skin nodule over the medial aspect of the right arm that had been present for eight months. During this period, she was treated with antibiotics by her general practitioner for a suspected skin infection on multiple occasions. According to her medical history, she underwent total thyroidectomy for PTC (follicular variant, pT2N0) without any adjuvant radioactive iodine (RAI) ablation 30 years prior to her visit. 

**Figure 1:**
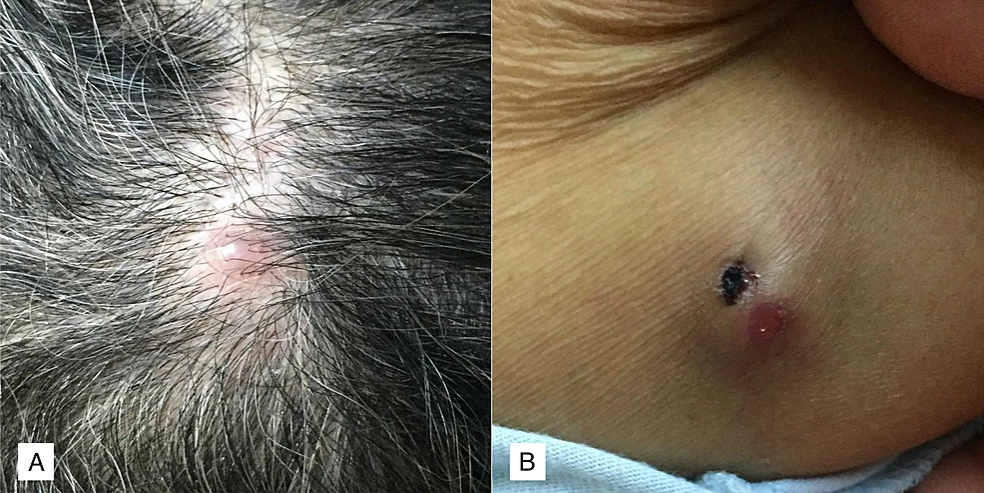
Erythematous nodule on the scalp (A) and the medial aspect of the right arm (B).

The patient was taking thyroxin 100 mcg daily and had irregular follow-ups with an endocrine clinic. During physical assessment, her performance status was an Eastern Cooperative Oncology Group (ECOG) score of 3. The scalp nodule was firm, erythematous, and mildly tender, with a size of 1 × 1 cm (Figure 1A). The other subcutaneous nodule over the medial right arm measured about 1.5 x 1.5 cm and was non-tender, mobile, and associated with erythema and scabbing (Figure 1B). The remaining clinical assessments were unremarkable. There was a high index of suspicion for skin metastasis as a differential diagnosis; therefore, punch biopsies were taken from both skin nodules in the clinic.

**Figure 2:**
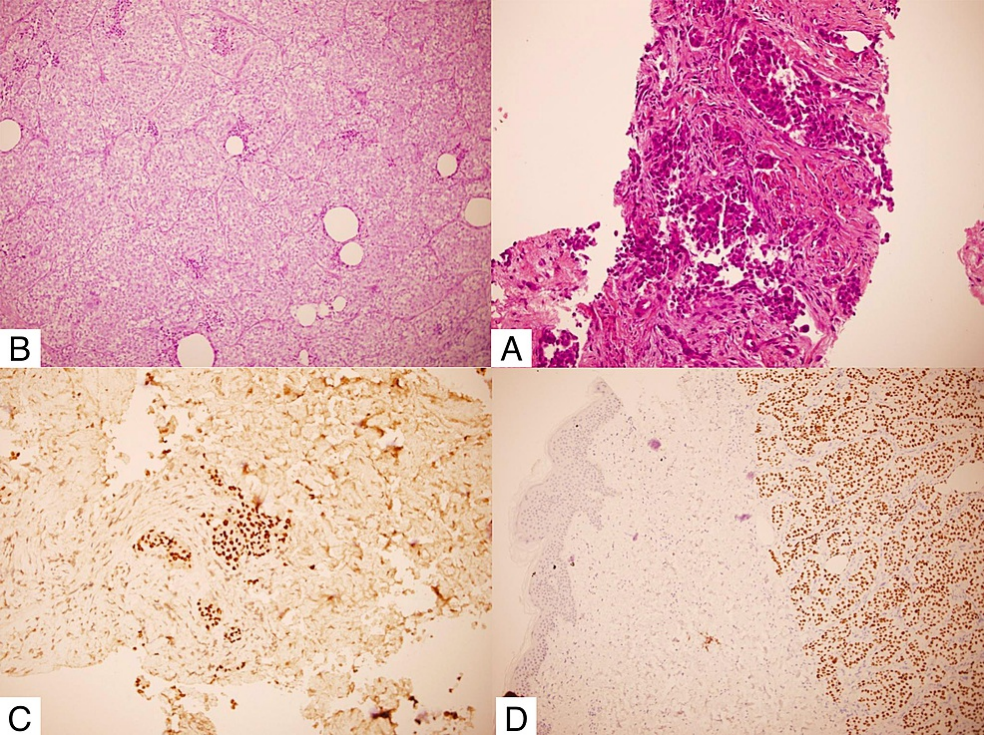
(A) Low-power view hematoxylin and eosin staining showing a papillary structure in the dermis. High-power view revealing (B) a fibrovascular core with papillary tumor cells containing large nuclei, nuclear grooves, and cytoplasmic pseudo-inclusions. Immunohistochemical staining positive for (C) thyroid transcription factor-1 and (D) thyroglobulin.

Hematoxylin and eosin staining showed a papillary structure in the dermis (low-power view; Figure 2A). The high-power view revealed a fibrovascular core with papillary tumor cells containing large nuclei, nuclear grooves, and cytoplasmic pseudo-inclusions (Figure 2B). Immunohistochemical staining was positive for thyroid transcription factor-1 (TTF-1) (Figure 2C) and thyroglobulin (Tg) (Figure 2D), which confirmed the diagnosis of skin metastasis originating from PTC. 

The serum thyroglobulin level was 3,431 μg/L and the anti-Tg antibody level was 127 IU/L. Staging workup was performed immediately after the diagnosis of skin metastasis. Neck ultrasound showed no local recurrence, and a diagnostic 131-iodine whole-body scan (WBS) revealed non-iodine avid disease. Computed tomography-positron emission tomography (CT-PET) revealed intense fluorodeoxyglucose avid lung and osseous metastases. Due to the non-iodine avidity, the patient was considered to be RAI refractory. She was started on low-dose sorafenib, an oral multikinase inhibitor, at a dose of 400 mg (200 mg twice a day) due to her poor performance status; she could not tolerate the medication after two cycles. The disease progressed over a period of time. After a multidisciplinary board meeting, the patient was transferred to palliative care to provide her with the best supportive care. The patient died of progressive systemic disease six months after the diagnosis of skin metastasis. 

## Discussion

The skin is a very rare site of metastatic involvement in PTC; there are only a few case reports in the English literature to date (Table 1) [3-5,7-17]. Skin metastasis might manifest as a pimple, slowly growing nodule, or rarely bleeding ulcer [16]. The median onset of skin metastasis from initial treatment for primary PTC was 8.25 years (range: 1-21 years), as shown in Table 1. Our patient presented with cutaneous metastasis 30 years after undergoing total thyroidectomy, which has not been described in any previous case report. In three previous case reports, synchronous skin metastases were documented at the time of newly diagnosed primary PTC [11,13,15].

**Table 1:** Previously published case reports of skin metastasis from papillary thyroid carcinoma. TT: total thyroidectomy; RAI: radioactive iodine; EBRT: external beam radiotherapy; nm: not mentioned; SD: stable disease; PD: positive disease

Case report	Age (y)/sex	Previous treatment	Location	Distant metastasis	Treatment	outcome	Follow-up
Kwon et al. [3]	55/female	TT & RAI ablation three years ago	Right neck subcutaneous nodule	No	Excision	Recurrence and was resected	Four months
Soylu et al. [4]	83/female	TT & RAI three years ago	Skin nodule right upper neck	No	Excision	nm	nm
Soylu et al. [4]	65/female	TT & RAI five years ago	Skin nodule left anterior neck	No	Excision	nm	nm
Farina et al. [5]	78/female	TT & RAI six years ago	Right parietal scalp nodule	Pancreas and bone	Excision/Sorafenib	SD	Two years (alive)
Farina et al. [5]	71/female	TT & RAI eight years ago	Left parietal scalp		Excision/RAI multiple times	Persistent high TG levels	Three years (/alive)
Farina et al. [5]	78/male	TT & RAI one year ago	Base of neck nodule		Excision/RAI multiple times	Nodal recurrence	Alive/PD
Sindoni et al. [7]	47/male	TT & RAI 11 years ago	Neck pimple-like lesions	No	Excision/RAI	nm	nm
Avram et al. [8]	63/male	TT & RAI 17 years ago	Purple skin nodule over left cheek	Lungs, lymph nodes, bones, and choroid	Excision/RAI multiple times	PD in skin and bones	nm
De Giorgi et al. [9]	86/male	TT & RAI 12 years ago	Skin nodule left supraclavicular region	Lungs	Excision	nm	nm
Shon et al. [10]	68/male	TT & RAI 21 years ago	Scrotal skin nodule	nm	nm	nm	nm
Heng et al. [11]	65/female	none	Left supraclavicular fossa	Lungs and lymph nodes	TT/EBRT	Died	Six months
Coulombe et al. [12]	68/female	TT	Thyroidectomy scar nodule	nm	Excision	nm	nm
Jehangir et al. [13]	65/female	none	Multiple scalp nodules	Bone	TT/resection	nm	nm
García-Gómez et al. [14]	69/female	TT	Right upper abdominal skin nodule	Lungs	nm	nm	nm
Camacho V, et al [15]	47/male	None	Nodule over nose	Lymph nodes	TT/RAI	nm	nm
Reusser NM, et al [16]	95/male	TT 9 y ago	Right anterior neck skin ulcer	nm	Multiple resections	Lost to follow-up	Twelve months
Lira MLA, et al [17]	55/female	TT 6 y ago	Left anterior neck skin nodule	nm	nm	nm	nm
Ronga G, et al [18]	59/female	TT 20 y ago	Neck skin nodule, on the surgical cicatrix	No	Excision	nm	nm
Elgart GW, et al [19]	59/male	Subtotal thyroidectomy & RAI 3 y ago	Parietal scalp	Left femur, right side of the chest, & both lungs	Excision	nm	nm
Bucerius J, et al [20]	57/female	right-sided hemithyroidectomy and left-sided palliative partially tumor resection, and RAI	Left proximal thigh, and left thorax	Choroid, lung, and lymph nodes	Resection	Died	56 months
Klonaris D, et al [21]	79/male	TT 16 years ago	Anterior thoracic wall skin	Muscles and bone	Excision	No clinical signs of recurrence	Nine months
Horiguchi et al [22]	62/male	Partial thyroidectomy three years ago	left temporal region of the scalp	Bone	Excision/RAI	Sternal metastasis, treated with external irradiation	nm
Horiguchi et al [22]	70/female	Tumor resection 1one year ago	Two on the frontal region of the head, multiple subcutaneous nodules of the abdomen, and extremities	nm	Largest tumor was excised, rest nodules were observed	Slow growth	nm
Doutre et al [23]	59/female	TT & RAI eight years and five months ago	Three firm nodules on the scalp	Bone and lungs	nm	Died	nm

Numerous mechanisms for the eventuality of skin metastasis from PTC have been postulated, including direct extension, hematogenous spread, lymphatic spread, and the implantation of exfoliated tumor cells during biopsy or thyroidectomy scars [12]. The majority of skin metastases are located in the scalp, face, and neck, which may be explained by the rich lymphatics and/or vascular supply of these regions that confine the cancer cell emboli from the circulation and provide a microenvironment for the successful proliferation of metastatic foci [16]. 

The treatment of skin metastasis from PTC varies according to the underlying systemic disease and iodine avidity. Isolated cutaneous metastasis can be cured with wide local excision; however, for skin metastasis with systemic disease, RAI, external beam radiation therapy, and targeted therapy (sorafenib) are the available recommended treatment options, with variable outcomes as shown in Table 1. Our patient had a non-iodine avid disease and was treated with low-dose sorafenib that she was poorly tolerant to. 

The prognosis is grave for patients with skin metastasis secondary to PTC with a high underlying tumor burden [11]. Our case is very rare as skin metastasis occurred 30 years after treatment for primary PTC, which has not been reported previously. Second, an extensive underlying non-iodine avid systemic disease survived for six months from the date of the diagnosis of the skin metastasis. 

## Conclusions

In conclusion, unusual skin nodules in patients with a history of thyroid malignancy should not be underestimated but should be addressed with a high index of suspicion of skin metastasis. A punch or excisional biopsy is advisable for a definitive diagnosis and prompt treatment. Earlier diagnosis can help identify the extent of disease recurrence and, therefore, to start the suitable line of treatment preventing further disease progression. Skin metastasis from PTC can present as a pimple, a slowly growing nodule, or even as a bleeding ulcer. It can present at any time; in our patient, it presented 30 years post primary cancer treatment. 
